# Diversity, Equity, and Inclusion Among Anesthesiology Trainees

**DOI:** 10.1089/whr.2021.0123

**Published:** 2022-04-07

**Authors:** Shyam Patel, Keldon K. Lin, Adam J. Milam, Soojie Yu, Gwendolyn Raynor, Khurmi Narjeet, Ricardo Verdiner, Marlene E. Girardo, Lopa Misra

**Affiliations:** ^1^ Mayo Clinic Alix School of Medicine, Scottsdale, Arizona, USA.; ^2^ Department of Anesthesiology & Perioperative Medicine, Mayo Clinic Arizona, Phoenix, Arizona, USA.; ^3^ Department of Quantitative Health Sciences, Mayo Clinic, Scottsdale, Arizona, USA.

**Keywords:** diversity, ethnicity, gender, gender identity, race, sexual orientation

## Abstract

**Background:**

Historically in medicine, women and minorities have been underrepresented. This trend is especially significant in the anesthesiology workforce.

**Objective:**

The goals of this study were to quantify the current state of diversity by race/ethnicity, gender, and sexual orientation among anesthesiology residents.

**Methods:**

An institutionally reviewed and validated survey was delivered through Qualtrics to 130 anesthesiology program directors. Topics addressed included gender identity, sexual orientation, racial and ethnic background, rationale for pursuing anesthesiology, and medical training experiences. The study was administered from February to April 2021; 135 anesthesiology residents responded to the survey.

**Results:**

The sample was 44.4% white (*n* = 60), 54.1% male (*n* = 73), and 83.7% (*n* = 113) of respondents self-reported as straight or heterosexual. Respondents indicated that role models/mentors were somewhat or very important in their desire to pursue anesthesiology (*n* = 85; 67.2%), 42% reported that having women/diverse faculty was somewhat or very important in their decision to pursue anesthesiology. Discrimination during the anesthesiology residency application process or as a resident ranged from 4.4% due to sexual orientation to 18.7% due to gender/gender identity and race/ethnicity.

**Conclusions:**

Experiences of discrimination based on race/ethnicity, gender, and gender identity continues to be a concern among anesthesiology trainees. Creating an environment that is inclusive and supportive of all trainees regardless of race/ethnicity, gender/gender identity, and sexual orientation is needed. Interventions and strategies to create an inclusive environment may improve diversity within anesthesiology.

## Introduction

Historically in medicine, women and minorities have been underrepresented; this is especially significant in the anesthesiology workforce.^1^ For example, during the 2018–2019 academic year, 47.9% of graduates from U.S. medical schools were women compared with 29.2% of Accreditation Council for Graduate Medical Education-accredited anesthesiology program graduates.^2^ Despite efforts to increase the representation of women and racial minorities in anesthesiology, the proportion of anesthesiologists who belong to these groups still lags behind that of the general population. In 2020, residents of American Indian/Alaska Native (0.7%), black/African American (6.0%), and Hispanic/Latino (7.4%) backgrounds accounted for <15% of all anesthesiology residents.^3^

To improve diversity among anesthesiology trainees and subsequently anesthesiologists, trainees must feel supported and welcomed.^4,5^ The benefit of a diverse workforce is well documented; increasing diversity among anesthesiologists has the potential to mitigate health care disparities and improve patient satisfaction.^6–9^ Consequently, many medical schools and residency programs have prioritized increasing diversity among matriculants. Although demographic data have been reported for anesthesiology residents to examine the diversity among the workforce, there is a paucity of information available regarding anesthesiology residents' experiences of discrimination based on gender, racial identity, or sexual orientation.^10^

The limited data on the influence of sexual orientation among medical trainees may result from sexual minorities' fear of discrimination and/or mistreatment.^11^ Prior studies have demonstrated a relationship between discrimination, peer connectedness, and mental health among African American medical students as well as differences in the prevalence of social support and connectedness as well as mental health symptoms by gender.^12,13^ Understanding experiences of discrimination among trainees may inform interventions, help improve mental health, and increase peer connectedness—again, we may be able to improve diversity if trainees feel supported and welcomed.

In addition to creating an inclusive environment, it is also important to examine factors motivating women and underrepresented minority students to select anesthesiology as a specialty in the face of perceived discrimination. Specialty selection generally depends on several factors, including lifestyle, work-life balance, and discipline interest.^14^ For example, one study found that medical graduates viewed salary and working conditions to be the main reasons for pursuing a residency in anesthesiology.^15,16^

However, mentorship in medicine is also critical for career development and advancement; relatedly, the lack of mentorship or discouragement from attending anesthesiologists to pursue anesthesiology may prevent certain demographics from entering the speciality.^17,18^ A survey of attending anesthesiologists found that 10% of female anesthesiologists would counsel a student against pursing anesthesiology based on the obstacles related to motherhood.^17^ Guidance from mentors serves the dual purposes of supporting professional growth and blunting the negative impact of workplace discrimination. Of note, underrepresented minorities in academic medicine have been reported to receive less mentoring compared with their nonminority peers.^19^

Accordingly, this study seeks to address gaps in the literature by (1) examining the motivating factors for choosing a career in anesthesiology, (2) quantifying the reported experiences of discrimination among anesthesiology residents during their training process, and (3) providing data to support future interventions to reduce the impact of discrimination among anesthesiology trainees.

## Methods

The survey was developed and reviewed by a team of five faculty anesthesiologists and two medical students at the Mayo Clinic Alix School of Medicine in Phoenix, AZ. Prior surveys performed in academic medicine on diversity, equity, and inclusion were reviewed to develop our survey instrument.^20,21^ The survey was screened by our institutional research and survey specialists (Mayo Clinic) for appropriate wording, terminology, and validation of questions relating to sexual orientation, race/ethnicity, and gender.

The survey questionnaire was also reviewed by our institutional committee on diversity, equity, and inclusion as well as our departmental research committee. The requirement for written informed consent was waived by the institutional review board at Mayo Clinic (Rochester, MN). Topics addressed in the survey included gender identity, sexual orientation, racial and ethnic background, factors for pursuing anesthesiology, and medical training experiences (Supplementary Appendix (SA).

The survey was electronically distributed through a password-protected Qualtrics account that was e-mailed to 130 anesthesiology program directors across the country to be distributed to their respective residents for completion. A written statement informing the program directors of the survey goals, approximate time to complete survey, and possible risks associated with participation was included in the e-mail. The survey was open for 8 weeks between February 2021 and April 2021. Three e-mail reminders were sent out to program directors during this period. Participation was voluntary, anonymous, and there was no compensation provided for completing the survey. IP addresses of survey responses were checked to prevent inclusion of duplicate responses.

Descriptive statistics were used to summarize questionnaire data and counts, and percentages were used for reporting survey responses. Chi-square test was used to compare students who experienced racial/ethnic or gender/gender identity discrimination by race/ethnicity and gender, respectively. *p*-Value <0.05 was considered statistically significant. Statistical analysis was performed using R version 3.6.2.

## Results

The majority of respondents self-identified as white (*n* = 60; 44.4%) followed by Asian (*n* = 27; 20.0%), multiracial/other (*n* = 23; 17.0%), Hispanic (*n* = 13, 9.6%), and black (*n* = 12, 8.9%); 83.7% identified as being straight/heterosexual, and 11.9% identified as lesbian, gay, bisexual, transgender, queer, (questioning), intersex, asexual, and (agender) (LGBTQIA) (Table 1). Slightly more than half of the respondents reported male gender/gender identity (*n* = 73, 54.1%) and 39.3% were first- or second-year residents (*n* = 53, 39.3%).

**Table 1.** Demographic Characteristics

Baseline characteristics	*n* (%)
Gender	
Female	58 (43.0)
Male	73 (54.1)
Nonbinary/third gender	2 (1.5)
Prefer not to answer/other	2 (1.5)
Race	
Asian (East/Southeast/Indian)	27 (20.0)
Black or African American	12 (8.9)
Hispanic or Latinx	13 (9.6)
Multiracial or other	23 (17.0)
White or Caucasian	60 (44.4)
Sexual orientation	
LGBTQIA	16 (11.9)
Straight or heterosexual	113 (83.7)
Prefer not to answer/other	6 (4.4)
Years in training	
Intern/transitional year/CA-0	21 (15.6)
CA-1	32 (23.7)
CA-2	49 (36.3)
CA-3	31 (23.0)
Fellow	2 (15.0)

*n* = 135.

CA, clinical anesthesiology; LGBTQIA, lesbian, gay, bisexual, transgender, queer, (questioning), intersex, asexual, and (agender).

### Factors for pursuing anesthesiology

The participants responded to six different statements regarding their decision to enter anesthesiology; role models/mentors were the most important factor selected by participants with 67.2% (*n* = 85) reporting that role models/mentors were somewhat or very important. About 61% (*n* = 82) reported that income expectations were somewhat or very important, 58.9% (*n* = 79) reported that work-life integration was somewhat or very important, and 41.7% (*n* = 56) reported that women/diverse faculty was somewhat or very important.

Females were more likely to report that women/diverse faculty was somewhat or very important compared with males (54.4% and 32.8%, respectively, *p* < 0.001); there was no statistical difference by between white and non-white respondents (43.2% and 40.0%. respectively, *p* = 0.125) (Table 2).

**Table 2.** Importance of Factors in Deciding to Pursue Anesthesiology

Factor	*n*-Miss		Least important		Neutral		Somewhat important		Very important		Most important	
	*n*	%	*n*	%	*n*	%	*n*	%	*n*	%	*n*	%
Role models/mentors	1	0.7	6	4.5	22	16.4	17	12.7	73	54.5	16	11.9
Work-life integration	1	0.7	1	0.7	10	7.5	5	3.7	74	55.2	44	32.8
Patient interaction	1	0.7	4	3.0	41	30.6	19	14.2	57	42.5	13	9.7
Income expectations	1	0.7	8	6.0	36	26.9	11	8.2	71	53.0	8	6.0
Opportunities for advancement	1	0.7	4	3.0	39	29.1	18	13.4	64	47.8	9	6.7
Women/diverse faculty	1	0.7	22	16.4	45	33.6	14	10.4	42	31.3	11	8.2

n-Miss represents the number of missing values from the survey question.

### Discrimination among anesthesiology trainees

The participants responded to experiences of discrimination due to racial or ethnic background, gender or gender identity, and sexual orientation. About 19% of trainees reported that they encountered discrimination during the residency application process because of their race/ethnicity, stratifying by white and non-white, 27% of non-white trainees, and 8.3% of white trainees reported they encountered discrimination (chi-square statistic = 8.8, *p* = 0.003). Similarly, 14.1% (*n* = 19) of trainees reported that their ethnic or racial background negatively impacted their experience as an anesthesiology resident or applicant including 3.3% of white respondents and 22.7% of non-white respondents (chi-square statistic = 10.3, *p* = 0.001).

Nearly a fifth of trainees reported encountering discrimination due to gender or gender identity (*n* = 25) including 35.1% of female respondents and 6.8% of male respondents (chi-square statistic = 16.4, *p* < 0.001); 4.4% of respondents reported that their gender identity negatively impacted their decision to pursue anesthesiology. The same percentage of trainees reported that they encountered discrimination due to sexual orientation (*n* = 6, 4.4%); 3.7% reported that their sexual orientation negatively impacted their decision to enter anesthesiology (Table 3).

**Table 3.** Discrimination Encountered During the Anesthesiology Residency Application Process or as a Resident

Discrimination	*n*-Miss		No		Yes		*p* ^*^
	*n*	%	*n*	%	*n*	%	
Due to racial or ethnic background							
Non-white (*n* = 75)	1	0.7	54	73.0	20	27.0	0.006
White (*n* = 60)	0	0.0	55	91.7	5	8.3	
Total (*n* = 135)	1	0.7	109	81.3	25	18.7	
Due to gender or gender identity							
Female (*n* = 58)	1	0.8	37	64.9	20	35.1	<0.001
Male (*n* = 73)	0	0.0	68	93.2	5	6.8	
Total (*n* = 131)	1	0.8	105	80.8	25	19.2	

n-Miss represents the number of missing values from the survey question.

## Discussion

As medical schools and national institutions are devoting efforts to increasing diversity in the medical workforce, we must ensure that trainees do not encounter discrimination based on race/ethnicity, gender, gender identity, or any other personal characteristic. Accordingly, this study sought to explore factors for pursuing anesthesiology and to understand experiences of discrimination among anesthesiology trainees.

Role models and mentors were the most important factor identified for entering anesthesiology. Relatedly, about half of respondents indicated that the presence of women and diverse faculty in anesthesiology were an important factor in pursuing this specialty. Mentorship may help to assuage the disproportionately negative impact of “otherness” experienced by residents who are underrepresented minorities, providing both logistical advice and strategies for coping with academic systems that frequently do not reflect successful physicians who look like them or share their cultural experiences.^22^

When deciding on orthopedic surgery residency rankings, the number of female orthopedic surgery residents and faculty were important when deciding on residency programs; furthermore, female applicants decided against programs with perceived sex biases.^21^ Similar concerns have been identified for female and minority applicants pursuing cardiothoracic surgery, and academic medicine broadly.^23,24^ Interventions to increase organizational support and leadership roles for women and others underrepresented in anesthesiology may reduce the burden of structural gender discrimination and increase workforce diversity.^25^

Experiences of discrimination were relatively common and there were differences by race/ethnicity and gender. Nearly a fifth of respondents reported experiencing discrimination based on their race/ethnicity. Racial and ethnic discrimination in medicine is an urgent problem and our data demonstrate the need for interventions to reduce experienced racial or ethnic discrimination among anesthesiology trainees. Furthermore, when the data were stratified by non-white and white trainees, there was a significantly higher proportion of non-white trainees that reported experiencing racial or ethnic discrimination and felt their racial/ethnic background negatively impacted their training experience.

This finding is consistent with prior studies that have shown that underrepresented minorities in medicine report higher rates of perceived discrimination.^13,26^ In addition, it has been reported that underrepresented minorities in medicine felt they had to navigate more challenges regarding cultural identity and professional identity due to their background.^27^ Perhaps this can be attributed in part to decreased mentorship, which is particularly critical for the professional development of underrepresented minorities.^19,27^

Alarmingly, more than a third of female respondents reported experiencing discrimination on the basis of their gender identity, which was significantly higher than what was reported by male respondents. This is consistent with a 2018 survey that found female anesthesiology residents perceive more gender-based discrimination at work.^28^ It has been reported that in anesthesiology, women occupy fewer leadership positions, receive fewer awards, and publish less frequently, indicating structural discrimination.^28,29^

This finding is an important factor that likely contributes to the gender inequality found in anesthesiology and relates to the lagging rates of women entering the field of anesthesiology.^2,19,28^ In addition, the role of motherhood during anesthesiology training has also been investigated and cited as a potential source for the decreased representation of women in the field.^17^

Sexual orientation remains a topic that is rarely discussed in the world of academic medicine and under-researched in the context of diversity, equity, and inclusion. There is a paucity of data regarding sexual orientation and experienced discrimination due to sexual orientation within anesthesiology. A recent study reported nonheterosexual anesthesiologists are more likely to report being made uncomfortable about their sexual orientation.^29^

Our study found discrimination due to sexual orientation among respondents to be much lower compared with experiences of racial/ethnic discrimination or gender discrimination. Further research in sexual orientation demographics and discrimination is needed not only in anesthesiology but also in academic medicine. There is little to no research on sexual orientation demographics in the literature currently, and future surveys should include questions related to sexual orientation to better understand how sexual orientation affects those who enter medical training.

There are several limitations that warrant discussion. First, there is no way estimate the response rate for the study. The survey was e-mailed to program directors who were responsible for e-mailing residents in their programs. We are unable to determine whether program directors received our e-mail regarding the study; furthermore, we are unable to ascertain if program directors e-mailed the survey to the residents in their program and if the residents opened the e-mail.

In addition, fatigue from online communication due to the pandemic has also likely contributed to the number of participants that responded to our survey. Even though survey respondents were assured that their responses were secure and anonymized, some respondents may have been hesitant to disclose certain aspects of their identity and experiences out of concern for personal or professional consequences.

As a result, survey answers may be skewed toward what respondents perceived as socially acceptable rather than their true feelings and experiences. The survey also allowed individuals to self-classify as LGBTQIA, a term that comprises sexual orientation, gender identity, and allyship; in retrospect, we appreciated that the breadth of this category and its variable interpretation may have obscured results. Finally, the results of our analysis are inherently limited by the simplistic nature of the available answer choices on the survey. Although survey respondents were asked to categorize experiences as positive or negative, in reality, an individual's experiences may be unique, complex, and not easily categorized as simply positive or negative.

## Conclusions

Despite these limitations, this study fills a gap in the literature by increasing our understanding of the factors that motivate trainees to enter anesthesiology and examining trainees' experiences of discrimination based on their race/ethnicity, gender, and sexual orientation. Future studies of the anesthesiology workforce are needed to assess the changes in demographic trends as it relates to diversity, equity, and inclusion. In addition, studies should examine inequity or discrimination on the basis of sexual orientation. Reducing experiences of discrimination for females and minority anesthesiology residents can increase diversity among anesthesiologists.

It is also important to understand these findings in the context of intersectionality, which describes how certain demographics or characteristics can compound themselves to increase risk; this may lead to increased burnout, depression, anxiety, and limited career growth. The intersection of non-white race/ethnicity, female gender, nonheterosexual orientation, being an anesthesiology resident, and perceptions of personal experiences as discriminatory all compound to create systemic barriers.

Despite these negative effects, opportunities for intervention are abundant and practical. Career workshops, mentor guidance, and peer networking groups are all essential to improving the diversity of the anesthesiology workforce and should continue to be implemented to increase access, exposure, and awareness.

## Ethical Approval

This study was deemed IRB exempt and approved on February 26, 2021 by the Mayo Clinic IRB.

## Supplementary Material

Supplemental data

## Supplementary Material

Supplementary Appendix SA

## Abbreviations Used

CA: clinical anesthesiology
LGBTQIA: lesbian, gay, bisexual, transgender, queer, (questioning), intersex, asexual, and (agender)
