# Supplementary material for: Diversity, Equity, and Inclusion Among Anesthesiology Trainees
Source: Womens Health Rep (New Rochelle). 2022 Apr 7;3(1):414–9. doi: 10.1089/whr.2021.0123 (PMC9081057; doi:10.1089/whr.2021.0123)
Supplement: Supplemental data [file Suppl_AppendixSA.docx]

**Appendix 1: Survey Disclosure Email**

1. Statement on project background and objectives of the survey

To date, little research has been performed that investigates the demographics and diversity of U.S. anesthesiologists or the factors that led them to enter anesthesiology. Our study’s primary aim is to investigate different aspects of diversity (e.g., ethnicity, sexual orientation, gender identity) in practicing U.S. anesthesiologists and how these factors contribute towards medical school graduates’ decision to pursue a career in anesthesiology. Our secondary aim is to see if personal preferences (e.g., lifestyle, career development) contributed to this decision as well.

1. Risks/benefits of participation (none)

There are no risks associated with participating in this survey, which will take approximately 3 minutes to complete. The primary benefit of participating in this survey is the opportunity to contribute to novel research that will identify and eventually address demographic and other barriers that U.S. anesthesiologists face in entering anesthesiology and in their professional training.

1. Statement stating participation is voluntary.

Participation in this survey is completely voluntary.

**Appendix 2: Survey Questions**

1. On a scale from 1 to 5 (with 1 being least important and 5 being most important), indicate the importance of the following factors in your decision to enter anesthesiology:

• Role models/mentors

• Work life integration

• Patient interaction

• Income expectations

• Opportunities for advancement

• Women/Diverse faculty

2. How many years ago did you graduate from medical school?

• Less than or equal to 5 years ago

• 6-10 years ago

• 10-15 years ago

• >15 years ago

3. How has your ethnic or racial background impacted your experience as an anesthesiology resident or applicant?

• Positively

• Negatively

• No impact

4. How has the ethnic or racial background of your co-applicants impacted your experience as an anesthesiology resident or applicant?

• Positively

• Negatively

• No impact

5. How has your sexual orientation impacted your decision to enter anesthesiology?

• Positively

• Negatively

• No Impact

6. How has your gender identity impacted your decision to enter anesthesiology?

• Positively

• Negatively

• No Impact

7. What is your gender identity?

• Male

• Female

• Transgender

• Non-binary/Non-conforming

• Prefer not to answer/Other (please describe if other)

8. What is your sexual orientation?

• LGTQIA

• Straight/Heterosexual

• Prefer not to answer/Other (please describe if other)

9. Which of the following best describes you? Select all that apply.

• Asian (East/southeast/Indian)

• Pacific Islander or native Hawaiian

• Black or African American

• Hispanic or Latinx

• Native American or Alaskan Native

• White or Caucasian

• Multiracial or Biracial

• Other (please describe):

10. Have you encountered discrimination during the anesthesiology residency application process due to the following factors (answer yes, no, or not applicable for each category):

• Racial or ethnic background _____

• Gender or gender identity_____

• Sexual orientation ____

11. Please check one of the following:

• I have a physical disability__

• I have a learning disability__

• I have both physical and learning disabilities__

• I have no diagnosed physical or learning disabilities__

12. My physical disability has impacted my decision to enter anesthesiology:

• Positively

• Negatively

• Not at all

• N/A

13. My learning disability has impacted my decision to enter anesthesiology:

• Positively

• Negatively

• Not at all

• N/A
